# Supplementary material for: Factors associated with the ability of adolescent girls and young women (AGYW) in sexual unions to negotiate for safer sex. An analysis of data from the 2018 Zambia Demographic and Health Survey (ZDHS)
Source: PLOS Glob Public Health. 2024 Sep 4;4(9):e0003614. doi: 10.1371/journal.pgph.0003614 (PMC11373833; doi:10.1371/journal.pgph.0003614)
Supplement: Attachment — Submitted filename: Response to reviewers.docx [file pgph.0003614.s001.docx]

TEEBENY ZULU,
THE UNIVERSITY OF ZAMBIA,
26^TH^ JULY, 2024.

Dear, Lucy Chimoyi Loyce, Kobusingye and Irene Mugenya,

I am writing to address the comments and feedback provided on my manuscript, *“Factors Associated with the Ability of Adolescent Girls and Young Women (AGYW) in Sexual Unions to Negotiate for Safer Sex. An Analysis of Data from the 2018 Zambia Demographic and Health Survey (ZDHS),”* submitted to ‘PLOS global public health’. I appreciate the time and effort taken to review my work and would like to respond to the points raised.

1. **Comment 1:** Are any of the AGYW in age-disparate relationships? if yes, what is the proportion and what is the mean age of the sexual partners
   - **Response**: There were no age-disparate relationships
2. **Comment 2:** The captions for the tables are not comprehensive enough and cannot pass the "fall to the ground test" What is being presented in the tables? The authors are advised to please revise these captions to include the population, outcome, setting and time period
   - **Response**: The captions on all the table have been rephrased to include the population, outcome, and setting and time period.
3. **Comment 3:** Table 2, is the term sexual behaviour or sexuality behaviour?

- **Response:** The term has been edited to sexual behaviour in table 2 and throughout the document

4. **Comment 4**. Table 1 included the place of residence variable, where a significant difference is observed between the two groups that make up the outcome. However, this variable is missing from the univariate and multivariate models. Can the authors explain this omission? Could it be that some of the provinces/regions where AGYW were less likely to negotiate sex are largely rural?

- **Response:** This variable was omitted because it was not statistically significant in the final model and it was not a priori

5. **Comment 5**. How was comprehensive HIV knowledge assessed?

- **Response:** Details of how comprehensive HIV/AIDS knowledge was assessed are presented in the studies [8-10] of the manuscript

6. **Comment 6**. Limitations are missing from the manuscript. Can the authors highlight some limitations?

- **Response: limitations of this study are presented together with its strengths in section 4.1**

7. **Comment 7**. Is there a variable that shows alcohol consumption by AGYW or inebriated sexual intercourse?

- **Response:** yes there is, very few AGYW aged 15-24 consumed alcohol as such this variable could not run in the model. Instead we looked at husband’s alcohol consumption our assumption was that this would trigger intimate partner violence which in turn would be associated with safer sex negotiation

8. **Comment 8:** Title has ignored ''refusal to have sex' as a major variable

- **Response:** Refusal to have sex is embedded in safer sex negotiation. See operational definitions in section 2.4

9. **Comment 9:** Abstract is clear and representative of the purpose, method, findings, conclusions and recommendations

- **Response:** Thank you

10. **Comment 10:** Introduction: Line 33 should be ''to negotiate condom'' not to ''request''. ...and or abstain from sex.....''

- **Response:** I have edited

11. **Comment 11:** define ''young women''

- **Response:** young women are females in the age rage 20-30, see operational definitions

12. **Comment 12:** after line 40, there is need for statistics of HIV prevalence among AGYW in Zambia (national statistics)

- **Response:** I have included the statistics in line 48

13. **Comment 13:** Lines 51-54: Provide literature on the role of soft skills such as self-esteem; self concept pillars such as self-efficacy; and other psychological contributors such as HIV risk perception

- **Response:** our study focused on the factors that affect the ability of AGYW to say no to sex or ask for condom use, hence we decided to focus on these

14. **Comment 14:** Lines 67-71: Indicate the study area and setting in terms of marital status because the age range is wide

- **Response:** our study population was all AGYW who were in sexual unions (married/cohabiting) hence we didn’t see the need to indicate marital status

15. **Comment 15:** Line 87: Show the Independent variable/s

- **Response:** independent variables are presented under study variables

16. **Comment 16:** Line 94: Knowledge of HIV , exposure to newspapers radio and TV, partners' alcohol consumption and IPV are not a sexual behaviour, rather, it is a non sexual attribute of HIV infection susceptibility.The sexual behaviours are are condom use and number of sexual partners (line 96)

- **Response:** you are right but we captioned them like that because they are linked to HIV infection susceptibility

17. **Comment 17:** * Operational definitions: Include who an adolescent is according to this study and what stage of adolescence this study focused on

- **Response:** we have included

18. **Comment 18:** Line 103:What attributes were the frequencies and percentages used to measure

- **Response:** to measure the proportion of safer sex negotiation among respondents

19. **Comment 19:** Line 125: Instead of ''most of them'', use, ''the majority''

- **Response:** noted and edited

20. **Comment 20:** Tables 1 and 2. Place the corresponding description for table one under table one. Do this for table two too

- **Response:** noted

21. **Comment 21:** After line 311: After the conclusions, suggestion mitigation strategies for low negotiation for safe sex and abstinence from sex among AGYW

- **Response:** noted thanks, though the approach we have taken for this paper was to give those suggestion in the closing stages of each paragraph in the discussion section

22. **Comment 22:** Line 38: For clarity, include "male counterpart."

- **Response:** included thanks

23. **Comment 23:** Line 42/43: The main area of study is negotiating for safer sex, which is one aspect of sexual autonomy. For clarity, consider expounding on what aspect of sexual autonomy is impacted.

- **Response:** edited thanks

24. **Comment 24:** Study Area and Setting: The justification for why we need this data for the Zambian population is not very clear. Consider including HIV prevalence in Zambia, particularly the prevalence in the AGYW age group. Additionally,since socioeconomic factors affect the main outcome consider discussing socioeconomic status, is there any linkage with residing in rural areas as this is a contributing factor to the inability to negotiate for safer sex practices.

- **Response:** thanks, statistics on HIV prevalence have been included in the introduction

25. **Comment 25:** Line 44 to 45: Little has been achieved in negotiating safer sex practices. Are there reasons for this? Link it to the need for understanding the factors associated with the inability to negotiate for safer sex.

- **Response:** yes. Reasons as well as the need to understand the factors associated with inability to negotiate for safer sex are given in the next paragraph line 47

26. **Comment 26:** Line 100 to 101: The operational definition of ANSS should be rephrased to make it clearer.

- **Response:** it has been rephrased, thanks

In conclusion, I hope that the revisions and clarifications provided meet your expectations. I am confident that these changes will enhance the manuscript and address the concerns raised. I look forward to your positive response and the opportunity to contribute to PLOS global public health..

Thank you for your consideration.

Sincerely,

TEEBENY ZULU

CONTACT: +260979598733/+260760884233

EMALE: teebenyzulu@gmail.com
