# Supplementary material for: Factors associated with the ability of adolescent girls and young women (AGYW) in sexual unions to negotiate for safer sex. An analysis of data from the 2018 Zambia Demographic and Health Survey (ZDHS)
Source: PLOS Glob Public Health. 2024 Sep 4;4(9):e0003614. doi: 10.1371/journal.pgph.0003614 (PMC11373833; doi:10.1371/journal.pgph.0003614)
Supplement: Attachment — Submitted filename: Response to reviewers.docx [file pgph.0003614.s002.docx]

TEEBENY ZULU,
THE UNIVERSITY OF ZAMBIA,
8^TH^ AUGUST, 2024.

Dear, Lucy Chimoyi Loyce,

I am writing to address the comments and feedback provided on my manuscript, *“Factors Associated with the Ability of Adolescent Girls and Young Women (AGYW) in Sexual Unions to Negotiate for Safer Sex. An Analysis of Data from the 2018 Zambia Demographic and Health Survey (ZDHS),”* submitted to ‘PLOS global public health’. I appreciate the time and effort taken to review my work and would like to respond to the points raised.

1. **Comment 1:** In the response, the authors clarify that there were no age-disparate relationships but did not mention the mean age of the male sexual partners. This is important because an older age (>5 years) has been known to impact the ability to negotiate sex for AGYWs.
   - **Response**: To measure age-disparate sex, we created a continuous variable whose values were the difference in age between the respondent and her husband/partner. This variable right-skewed with mean 4.98 years suggesting no significant age-disparate relationships, with the mean age of sexual partners estimated at 26 years
2. **Comment 1:** In the table showing "Sexual characteristics...." and the paragraph showing these results, i wonder why variables such as watching TV, reading newspaper etc are included. Can the authors revise the titles of the caption and paragraph or create a separate table and paragraph for these results? It is not clear why exposure to TV, radio and newspapers can be considered as a sexual behaviour trait (this is mentioned in the methods section)
   - **Response**: we have changed the caption to ‘behavioural characteristics’
3. **Comment 1:** The response on how comprehensive HIV knowledge was assessed is not convincing. Was comprehensive HIV knowledge a stand alone variable in the DHS dataset or a composite variable generated from a series of questions in the data? If the latter, i would expect the authors to explain how this was arrived at in the methods section, similar to the ANSS definition.
   - **Response**: Comprehensive knowledge on HIV/AIDS was a composite variable’. It was defined as correctly knowing two ways to prevent HIV transmission and rejection of the three most common misconceptions about HIV. This variable was measured by asking each woman whether or not she agreed with the following five statements: (1) Consistent use of condoms during sexual intercourse can prevent HIV transmission; (2) Limiting sex to just one uninfected faithful partner can prevent HIV transmission; (3) A healthy-looking person can have HIV; (4) A person can get HIV through mosquito bites; (5) A person can get HIV by sharing food with an HIV-infected person. A respondent was considered to have comprehensive HIV/AIDS knowledge if he/she correctly responding to all the five questions and a code ‘Yes’=1 was assigned, otherwise a ‘No’=0 was given.
4. **Comment 4:** Please review your reference list to ensure that it is complete and correct. If you have cited papers that have been retracted, please include the rationale for doing so in the manuscript text, or remove these references and replace them with relevant current references. Any changes to the reference list should be mentioned in the rebuttal letter that accompanies your revised manuscript. If you need to cite a retracted article, indicate the article’s retracted status in the References list and also include a citation and full reference for the retraction notice

- **Response:** we have reviewed references and we haven’t found any retracted articles

In conclusion, I hope that the revisions and clarifications provided meet your expectations. I am confident that these changes will enhance the manuscript and address the concerns raised. I look forward to your positive response and the opportunity to contribute to PLOS global public health.

Thank you for your consideration.

Sincerely,

TEEBENY ZULU

CONTACT: +260979598733/+260760884233

EMALE: teebenyzulu@gmail.com
